# Electrochemical preparation system for unique mesoporous hemisphere gold nanoparticles using block copolymer micelles†

**DOI:** 10.1039/d0ra01072c

**Published:** 2020-02-28

**Authors:** Hyunsoo Lim, Tomota Nagaura, Minjun Kim, Kenya Kani, Jeonghun Kim, Yoshio Bando, Saad M. Alshehri, Tansir Ahamad, Jungmok You, Jongbeom Na, Yusuke Yamauchi

**Affiliations:** Australian Institute for Bioengineering and Nanotechnology (AIBN) and School of Chemical Engineering, The University of Queensland Brisbane QLD 4072 Australia y.yamauchi@uq.edu.au; Key Laboratory of Eco-chemical Engineering, College of Chemistry and Molecular Engineering, Qingdao University of Science and Technology Qingdao 266042 China; Department of Chemistry, Kookmin University 77 Jeongneung-ro, Seongbuk-gu Seoul 02707 Republic of Korea; International Center for Materials Nanoarchitechtonics (MANA), National Institute for Materials Science (NIMS) 1-1 Namiki Tsukuba Ibaraki 305-0044 Japan; Institute of Molecular Plus, Tianjin University No. 92 Weijin Road, Nankai District Tianjin 300072 P. R. China; Australian Institute of Innovative Materials (AIIM), The University of Wollongong Squires Way North Wollongong NSW 2500 Australia; Department of Chemistry, College of Science, King Saud University Riyadh 11451 Saudi Arabia; Department of Plant & Environmental New Resources, Kyung Hee University 1732 Deogyeong-daero, Giheung-gu Yongin-si Gyeonggi-do 446-701 South Korea

## Abstract

Gold nanoparticles (AuNPs) are widely used in various applications, such as biological delivery, catalysis, and others. In this report, we present a novel synthetic method to prepare mesoporous hemisphere gold nanoparticles (MHAuNPs) *via* electrochemical reduction reaction with the aid of polymeric micelle assembly as a pore-directing agent.

## Abstract 2

Mesoporous hemisphere Au nanoparticles using self-assembled micelles, for the first time, are demonstrated by using electrochemical reduction on a Ti substrate.

## Graphical abstract

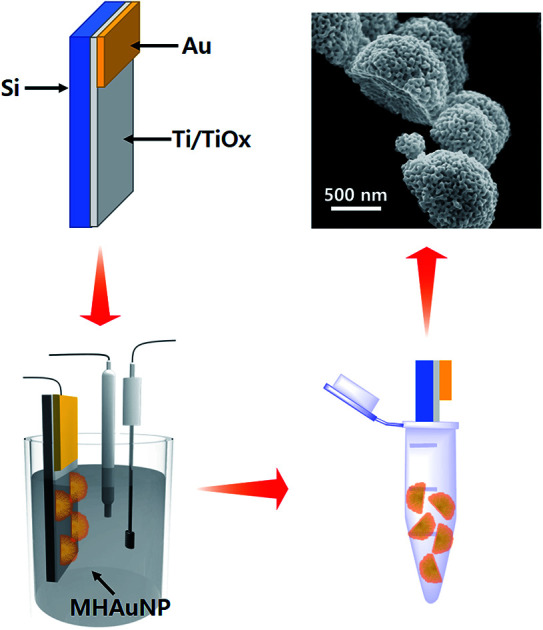

Gold (Au) is one of the most stable and versatile elements utilized in various fields, including catalysis, optics, and industrial purposes. Consequently, various shapes and sizes of AuNPs have been intensively studied to improve the performance of Au in different applications.^1–6^ Previously, nanoporous or dendritic metal nanostructures, including Au nanostructures, have been synthesized by employing different reagents and conditions such as SH-terminated amphiphilic surfactant,^7^ pH controlling,^8^ and hard-templates.^9,10^ The reported porous and dendritic Au nanostructures possess high surface areas and rich active sites, which in turn lead to highly enhanced catalytic activities.

Recently, a soft-template method using self-assembled micelles or lyotropic liquid crystals as pore-directing agents has allowed the successful synthesis of mesoporous nanoparticles^11–13^ and films^14–17^ with different metal compositions. The metals with mesoporous structures demonstrate superior catalytic activity per weight or surface area over their nonporous bulk forms. Previously, our group reported a several-fold increase in the catalytic activity of mesoporous metals in reactions such as the methanol oxidation reaction (MOR),^14,15^ ethanol oxidation reaction (EOR),^13,15–17^ and nitric oxide reduction^12^ as compared to their bulk nanoparticles and films. Such improvement in the catalytic activity of mesoporous structures is mainly attributed to their significantly larger surface areas, more exposed catalytically active sites, and increased durability against aggregation.

Interestingly, nanoporous or mesoporous Au structures had been successfully synthesized by using a dealloying method^18^ and a hard templating method.^9^ Such methods, however, are a little complicated, and pore-directing templates often remain within the pores, thus leading to severe contamination. Using a thiol group is an alternative way to synthesize mesoporous Au nanospheres.^7^ A significant drawback of using a thiol group, however, is its strong chemical bonding with Au, thus becoming unable to be removed. The synthesis of mesoporous structures using self-assembled polymeric micelles as soft-templates, on the other hand, is a more facile method with fewer synthetic steps, and it is also known to be free of contaminations within the pores. Although a soft-templating method using polymeric micelles has been utilized for the preparation of mesoporous Au and Au-based alloy films towards surface-enhanced Raman scattering (SERS) signals,^19^ glucose sensing,^20,21^ and MOR,^22^ the obtained morphologies have been limited to only films.

Despite such apparent benefits arising from mesoporous structures and their synthesis using soft-templates, the synthesis of mesoporous AuNPs using soft-templates has not been achieved yet. It is mainly due to the physical and chemical properties of Au which make it extremely hard to form mesoporous structures. Herein, we adopt an electrochemical approach and the soft-template method to synthesize MHAuNPs successfully. As discussed above, we expect MHAuNPs to be highly efficient in various applications in medical diagnosis,^23^ optical sensing,^24^*etc*.

In this report, MHAuNPs with different shapes and sizes are for the first time reported by changing various electrochemical deposition conditions such as applied potentials between electrodes and deposition times. Scheme 1 shows the schematic illustrations of the entire process of precursor preparation (Scheme 1a) and the MHAuNPs fabrication process (Scheme 1b), including the deposition and the detachment of the nanoparticles. The characterization methods implemented in this paper are mentioned in ESI.†

**Scheme 1:**
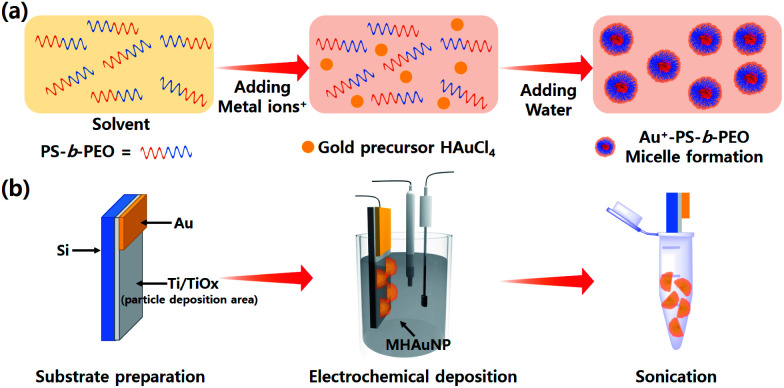
(a) The process of Au precursor solution preparation and (b) fabricating MHAuNPs by electrochemical reduction.

In a typical experiment, a p-doped silicon (Si) wafer was cleaned by using acetone, isopropyl alcohol, and deionized water (DIW) with sonication for 5 minutes, followed by nitrogen (N_2_) gas blowing to dry the Si wafer. After the wet cleaning process, the Si surface was treated by oxygen (O_2_) plasma for 5 minutes (Oxford Instruments PlasmaPro 80 Reactive Ion Etcher) to remove residual organic impurities. Then, 10 nm of titanium (Ti) layer and 100 nm of Au layer were deposited sequentially by electron beam evaporation (Temescal FC-2000 e-beam evaporator) at 10^−6^ torr. Commercially available Au etchant (Sigma-Aldrich) was used to etch the Au film to expose the Ti area (the left image in Scheme 1b). During etching, about 20 percent of Au area was left to be connected to the electrochemical work station, as drawn in Scheme 1b. In preparation of the Au precursor solution, 5 mg of poly(styrene)-*block*-poly(ethylene oxide) (PS-*b*-PEO, the number of average molecular weight (*M*_w_) for each block is 18 000 for PS and 7500 for PEO, respectively) was mixed in 1.5 ml of tetrahydrofuran (THF) followed by stirring at 300 rpm for 8 hours. Then, 0.75 ml of ethanol, 0.5 ml of HAuCl_4_ aqueous solution (40 mM), and 1.25 ml of DIW were added sequentially. The solution was stirred for another 30 minutes at 200 rpm. The existing block copolymer micelles can be confirmed by TEM observation, and the average diameter is 25 nm, as shown in Fig. S1.† For the electrochemical deposition, an electrochemical workstation (CH Instruments Inc. 660e) with three electrode system was used to deposit MHAuNPs on the Ti/Si substrate. After the deposition, the particles were carefully washed by chloroform, followed by a rinse using DIW to remove the residual micelles completely. To detach and collect MHAuNPs from the Ti/Si substrate, the substrate was soaked in ethanol and strongly sonicated for a few minutes (Scheme 1b).

Fig. S2† shows the details of the growth mechanism of MHAuNPs by different deposition times. At the initial stage (Fig. S2a†), small nanoparticles are generated by reducing Au ions in the precursor solution throughout the substrate. Then, the seed starts growing and forming MHAuNPs as the deposition time increases (Fig. S2b–e†). This similar growth mechanism is the same as the previous report.^19^ The high-angle annular detector dark-field scanning transmission electron microscopy (HAADF-STEM) image (Fig. S2f†) shows the mesopores inside the MHAuNPs are homogeneously generated. As-obtained MHAuNPs consist of a pure Au element without any impurities, as shown in Fig. S3.†

Fig. 1 and S4† show scanning electron microscope (SEM) images of MHAuNPs deposited at different voltages from −0.2 V to −0.9 V *vs.* Ag/AgCl at high magnification and low magnification, respectively. Different deposition voltages lead to significant changes in the particle sizes but slight differences in the particle shapes. The size distributions of MHAuNPs and the plots of the average diameters of MHAuNPs by different deposition voltages are described in Fig. 2. The distribution graphs show the large sizes of particles, such as more than 1 μm in diameter, when the high voltage (−0.2 V *vs.* Ag/AgCl) is applied (Fig. 2a). The distribution becomes narrower upon the lower applied voltage. The average diameter-applied voltage plots in Fig. 2b show that the average particle size decreases from around 1.1 μm at −0.2 V to about 300 nm at −0.9 V. Thus when the lower deposition voltages are applied (*i.e.*, the deposition rate is higher) (Fig. 1g–h), the smaller particles with a higher degree of size uniformity are obtained. The opposite trend is observed at higher deposition voltages (*i.e.*, the deposition rate is lower) (Fig. 1a and b), at which the particles become larger and their size uniformity decreases. This trend is because the higher voltage allows only a limited number of seed particles to be deposited on the Ti/Si substrate, and each seed individually grows with no additional seed formation. Whereas the lower voltage can allow a higher number of seeds, leading to a uniform supply of electrons from the working Ti/Si electrode (Fig. S5†). In addition, the lower deposition voltages make the particle shape more hemispherical in Fig. 1f–h.

**Fig. 1:**
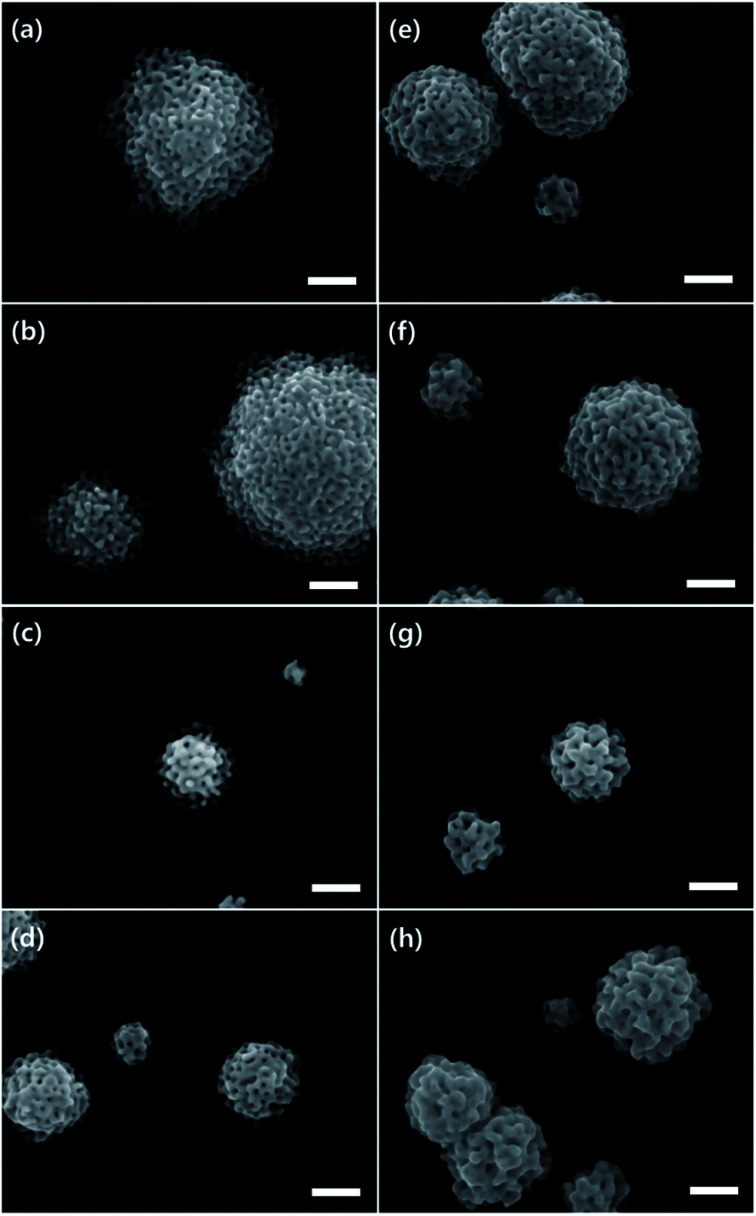
The SEM images of MHAuNPs electrochemically deposited at (a) −0.2 V, (b) −0.3 V, (c) −0.4 V, (d) −0.5 V, (e) −0.6 V, (f) −0.7 V, (g) −0.8 V, and (h) −0.9 V for 500 s. The scale bars indicate 200 nm.

**Fig. 2:**
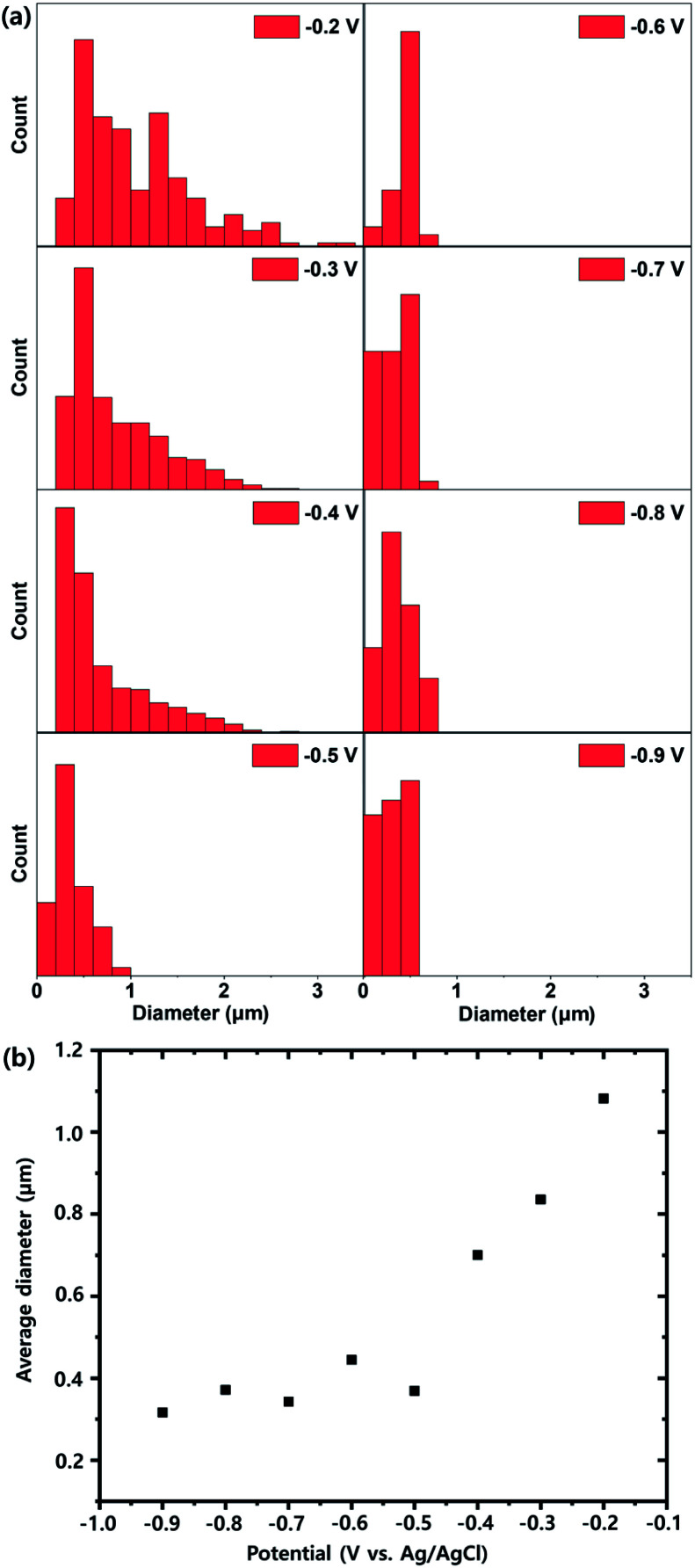
(a) Size distributions of MHAuNPs generated by different voltages and (b) the average diameter–the applied voltage plots.

Fig. 3 shows the SEM images of MHAuNPs deposited at −0.2 V and for different deposition times from 250 s to 1000 s. Although longer deposition time does not change the number of MHAuNPs, it leads to the growth of MHAuNPs in lateral and vertical directions. Although the MHAuNPs grow more than about two or three times larger at long deposition time, the mesoporous formation does not seem to be changed, as shown in insets in Fig. 3. This point indicates that the deposition time is not the main factor affecting the formation of mesoporous structures as well as the number of particles (seeds), but it affects the sizes of particles.

**Fig. 3:**
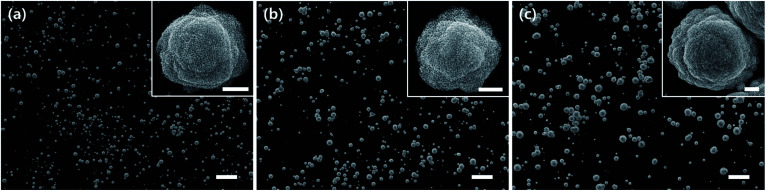
The SEM images of MHAuNPs deposited at −0.2 V (*vs.* Ag/AgCl) for (a) 250 s, (b) 500 s, and (c) 1000 s. The scale bars indicate 10 μm. The insets in each figure are magnified SEM images of each condition (The scale bars in insets indicate 500 nm).

In this report, 10 nm Ti layer on Si wafer plays an important key role in the formation of MHAuNPs, as previously mentioned in the experimental procedure. The use of the Ti substrate with low conductivity (*ca*. 2.38 × 10^6^ S m^−1^), which is about only 5.8% in comparison with that of Au (*ca*. 4.10 × 10^7^ S m^−1^), is not common in the electrochemical plating research field.^25–30^ Most of the papers on mesoporous metal structures synthesized by electrochemical deposition have utilized Au or Pt substrates due to its chemical stability and high electrical conductivity.^14–17^ Fig. S6† shows the amperometry (*i*–*t*) curves during the deposition of MHAuNPs (black dots) on a Ti/Si substrate and mesoporous gold films (red dots) on an Au substrate at the same deposition condition. As shown in Fig. S6,† around 1/7 times less current flows on the Ti/Si substrate throughout the deposition time. This low current density on the Ti/Si substrate is one of the factors for fabricating MHAuNPs. Low current density causes the formation of a few particles (*i.e.*, seeds) at the initial stage of the deposition and leads to seed growth in a few places, as explained in Fig. S2.† Furthermore, the use of Ti/Si substrates affects the bottom parts of MHAuNPs to become an arch shape. Only edges of MHAuNPs attach onto the Ti/Si substrates, as shown in Fig. 4. This attachment is because the interaction between the deposited MHAuNPs and the Ti substrate surface (probably, the Ti surface can be partially oxidized, forming TiO_*x*_) is very weak. Therefore, the deposited MHAuNPs can be easily detached from the Ti/Si substrates by sonicating the substrates in solvents (Scheme 1b). The collected MHAuNPs in a solvent are obtained as colloidal particles as shown in Fig. S7.† Such interesting hemispherical mesoporous nanoparticles have advantages to electrocatalytic activities in comparison to spherical mesoporous metals.^31^ The method using a Ti/Si substrate as a working electrode can be repeatedly implemented with one substrate and without change of the precursor solution, thus it can be effective for mass production in the future.

**Fig. 4:**
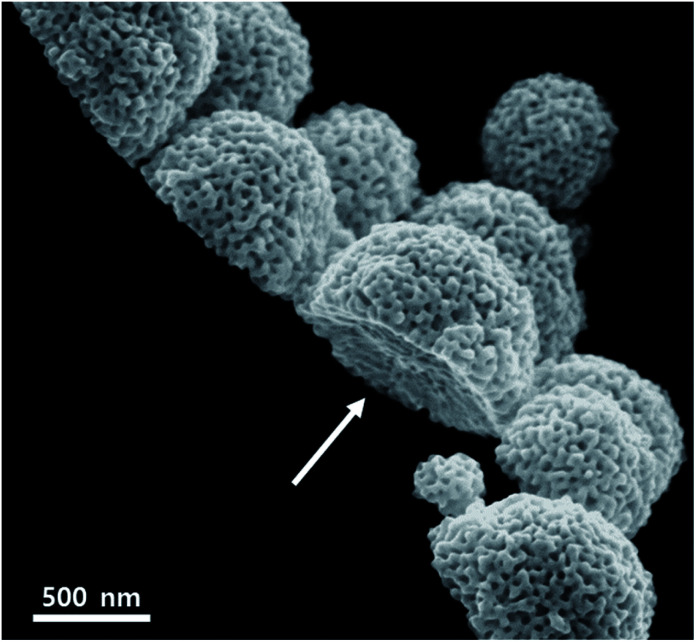
The SEM image of MHAuNPs deposited at −0.6 V. The arrow shows that the bottom of the MHAuNPs is an arch.

Finally, surface-enhanced Raman scattering (SERS) effects on MHAuNPs were investigated by using an adsorbate called rhodamine 6G (R6G), as shown in Fig. S8.† The resulting MHAuNPs at all conditions (−0.3 V, −0.6 V, and −0.9 V) show substantially strong SERS intensity (Fig. S8a†), while Ti/Si and Si substrates without MHAuNPs show noise level of intensity. To further investigate enhancement factor (EF) and limit of detection (LoD), various concentrations of R6G with MHAuNPs fabricated at −0.9 V were used for the SERS studies (Fig. S8b†). The main peak of SERS is 1363 cm^−1^, and it disappears from less than 10^−6^ M concentration, while the 1183 cm^−1^ peak still exists at 10^−8^ M (Fig. S8c†). The maximum EFs at 1363 cm^−1^ (10^−6^ M) and 1183 cm^−1^ (10^−8^ M) are 1.5 × 10^4^ and 3.1 × 10^6^, respectively (Fig. S8d†). Transmission electron microscope (TEM) images in Fig. S9† show the detailed particle structures and the electron diffraction (ED) pattern confirmed the crystal structure is the face-center cubic (FCC) structure. The sharp surface structures and the pores on MHAuNPs provide abundant hot spots that have been reported as the origin that enhances SERS intensity owing to the plasmon resonances.^19,32^ Besides, the high density of small-sized MHAuNPs (Fig. S5 and S10†) boosted higher SERS intensity.

In conclusion, we have synthesized MHAuNPs by using 10 nm Ti-coated Si substrates as a working electrode on a Si wafer and electrochemical deposition using self-assembled polymeric micelles as pore-directing agents. The low current generates Au seeds at only a few places, and it acts as the points that MHAuNPs start growing. The particle shapes and sizes can be controlled by changed applied voltages and deposition times. The lower voltages make small particles and the great hemispherical AuNPs with mesoporous architecture. The long-time deposition does not affect any mesoporous formation, but the particle shape and size. Besides, the low affinity between Au and Ti (probably, oxidized layer) results in the arch on the bottom of MHAuNPs, which helps the particles detached from the substrates easily. These results indicate that different thicknesses and compositions of working electrodes can provide different metal deposition phenomena, which can bring out unique shaped particles with mesoporous architectures in the future.

## Conflicts of interest

There are no conflicts to declare.

## Supplementary Material

RA-010-D0RA01072C-s001
