# Supplementary material for: Electrochemical preparation system for unique mesoporous hemisphere gold nanoparticles using block copolymer micelles
Source: RSC Adv. 2020 Feb 28;10(14):8309–13. doi: 10.1039/d0ra01072c (PMC9049955; doi:10.1039/d0ra01072c)
Supplement: RA-010-D0RA01072C-s001 [file RA-010-D0RA01072C-s001.pdf]

Electronic Supplementary Material (ESI) for RSC Advances.
This journal is © The Royal Society of Chemistry 2020

Characterization

Scanning Electron Microscope (SEM) and Energy-dispersive X-ray spectroscopy (EDS). The prepared MHAuNPs on the Ti/Si substrate are attached to an aluminum SEM sample mount by using carbon tape. To obtain SEM images of MHAuNPs, a field emission scanning electron microscope (FE-SEM) with a hot electron gun was used with 15 kV driving voltage. EDS with two X-ray detectors was conducted in the same instrument without air exposure.

Transmission Electron Microscope (TEM). MHAuNPs on the Ti/Si substrate were soaked in a centrifuge tube with ethanol and sonicated for 10 minutes to detach MHAuNPs from the substrate. The MHAuNPs-dispersed solution was washed three times and dropped on a holey carbon TEM grid. To obtain TEM images of MHAuNPs, the Hitachi HT7700 TEM instrument was used with 120 kV driving voltage.

Scanning Transmission Electron Microscope (STEM). The same sample as TEM measurement was used for STEM. For the STEM images, Hitachi HF5000 Cs-STEM/TEM instrument was used with 200 kV driving voltages.

Surface-enhanced Raman Scattering (SERS). For SERS measurement, different five types of substrates are prepared (*p*-doped Si wafer, 10 nm thick Ti substrate, MHAuNPs deposited at -0.3 V, -0.6 V, and -0.9 V on a Ti substrate). 100 μM rhodamine 6G (R6G) solution in ethanol was prepared by diluting a higher concentration (100 mM) of the solution. The R6G solution was drop-casted on the substrates and dried in air. For the SERS measurement, Renishaw Raman microscope system with a 785 nm excitation laser was used. The fluorescent curves are removed by using multi-polynomial fitting and the algorithm (J. Zhao, H. Lui, D. I. Mclean and H. Zeng, *Appl. Spectrosc.*, 2007, **61**, 1225.).

Micelle distribution measurement. Software called "ImageJ" was used to analyze micelle size distribution (**Fig. S1a**). The method uses the imaging process function by defining the circularity and adjusting the threshold of an image **(Imaging process step in Fig. S1a)**. After defining the parameters, the area of each micelle can be calculated **(Pore extraction step in Fig. S1a)**. Manual measurement function can be also available on the software when the number of samples (micelles) is not an enormous amount.

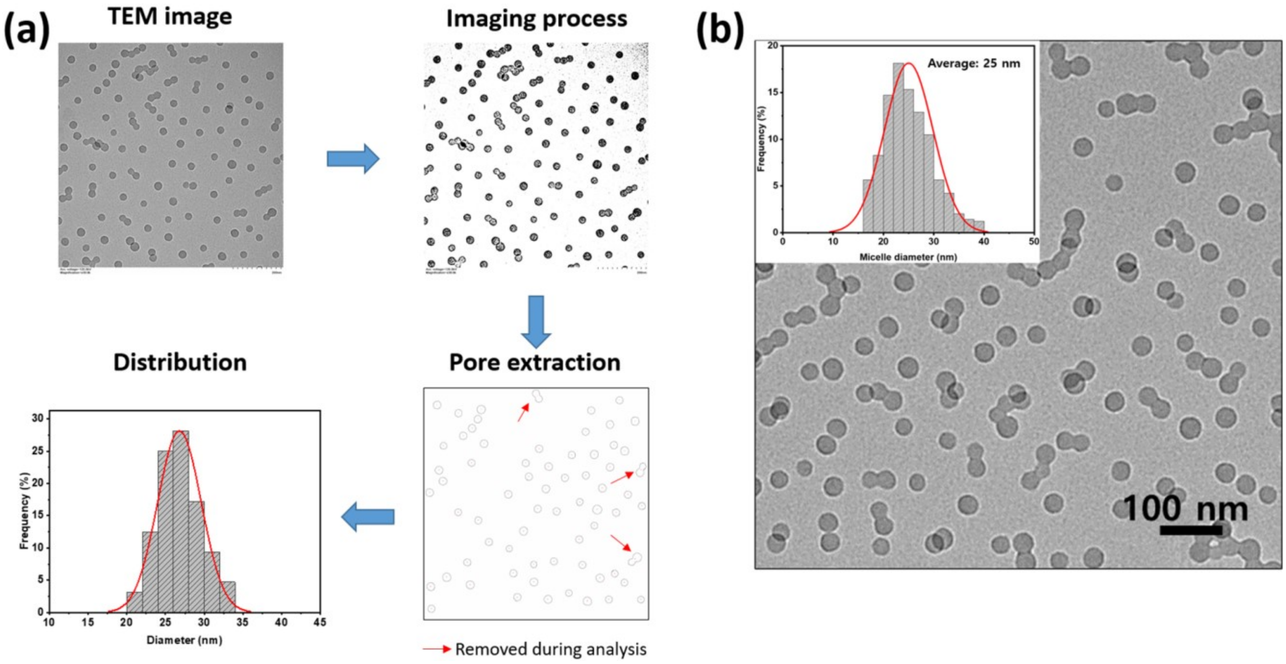

Fig. S1 (a) Scheme of the imaging processes using ImageJ for analyzing micelle distribution. (b) The TEM image of PS-*b*-PEO micelles used for MHAuNPs. The inset is the distribution curve of the micelles and the average diameter is 25 nm.

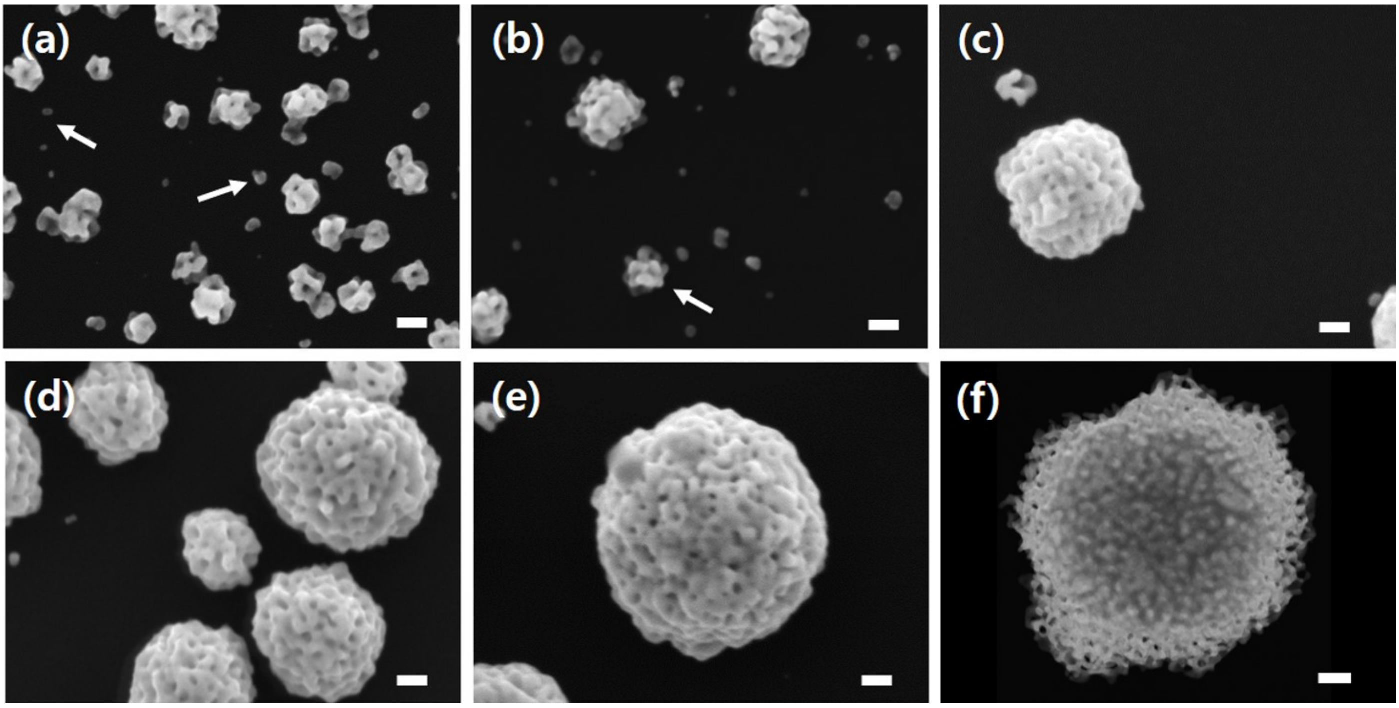

Fig. S2 SEM images of MHAuNPs deposited at -0.6 V for (a) 50 s, (b) 100 s, (c) 250 s, (d) 500 s, and (e) 1000 s, respectively (The scale bars indicate 100 nm). The arrows in the figures indicate an initial gold seed with mesopores. (f) The HAADF-STEM image of the MHAuNP deposited at -0.6 V for 500 s.

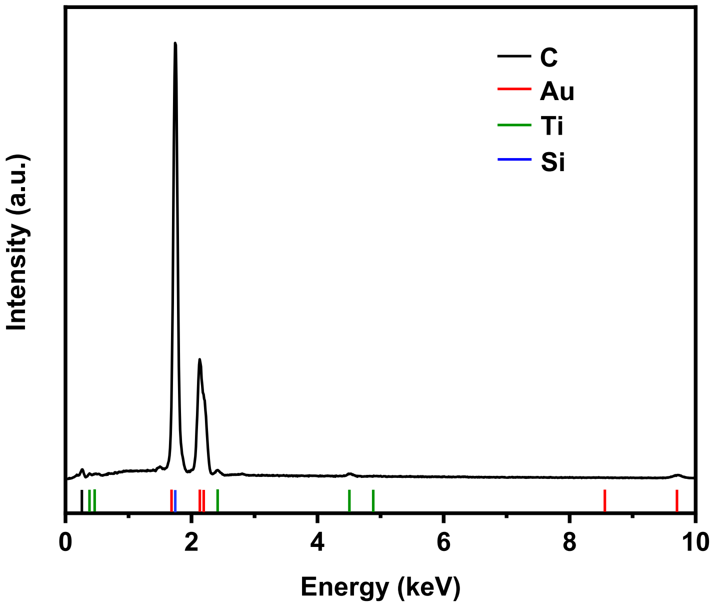

Fig. S3 Energy-dispersive X-ray spectra of MHAuNPs on a Ti/Si substrate.

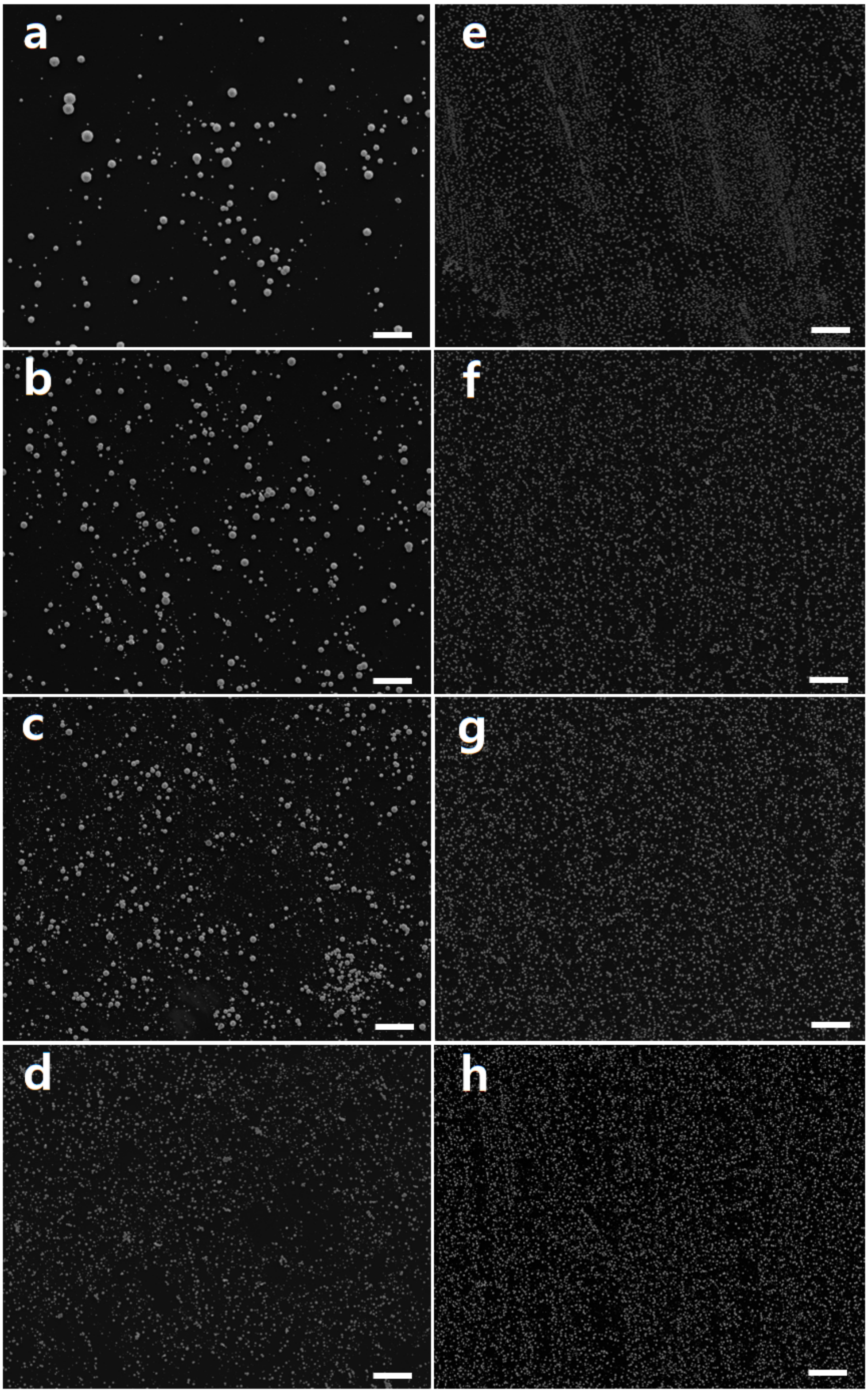

Fig. S4 Low-magnified SEM images of MHAuNPs electrochemically deposited at (a) -0.2 V, (b) -0.3 V, (c) -0.4 V, (d) -0.5 V, (e) -0.6 V, (f) -0.7 V, (g) -0.8 V, and (h) -0.9 V for 500 s. The scale bars indicate 10 μm.

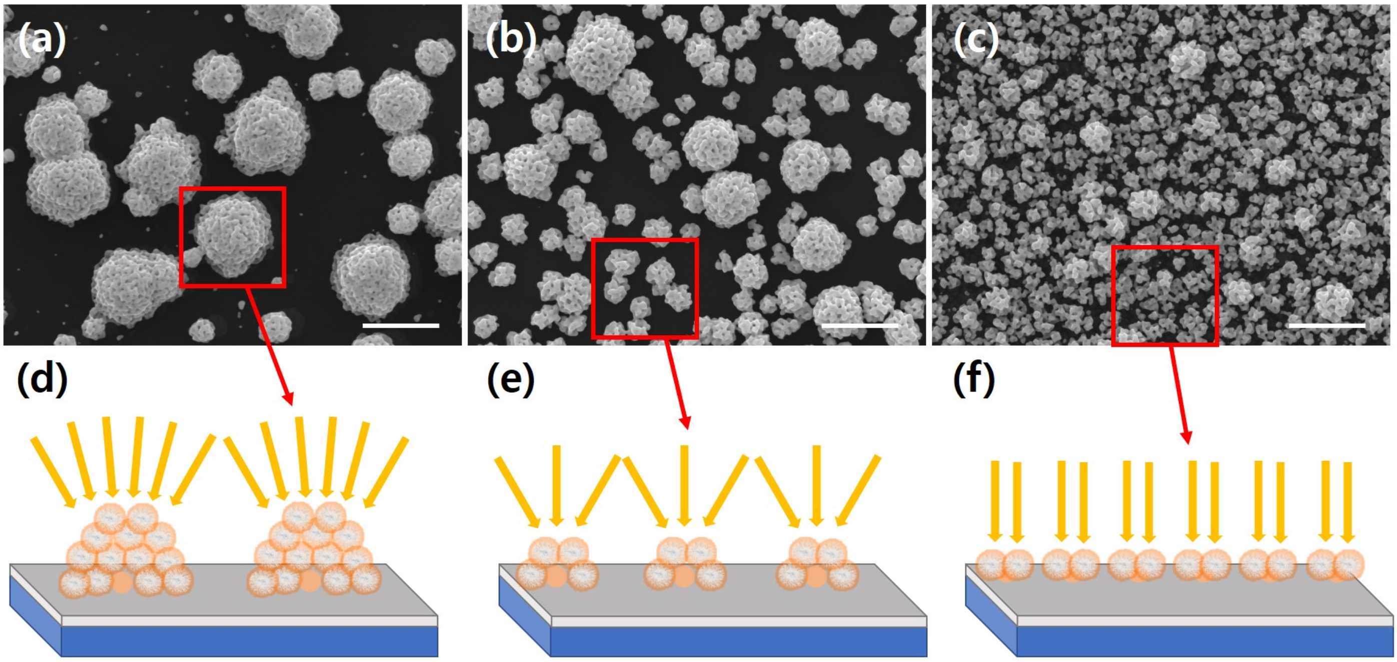

Fig. S5 SEM images of MHAuNPs deposited at (a) -0.3 V, (b) -0.6 V, and (c) -0.9 V for 1000 s. (d-f) The illustrated images of different MHAuNPs' formations by different applied voltages. The scale bars in Fig. S4a-c indicate 200 nm.

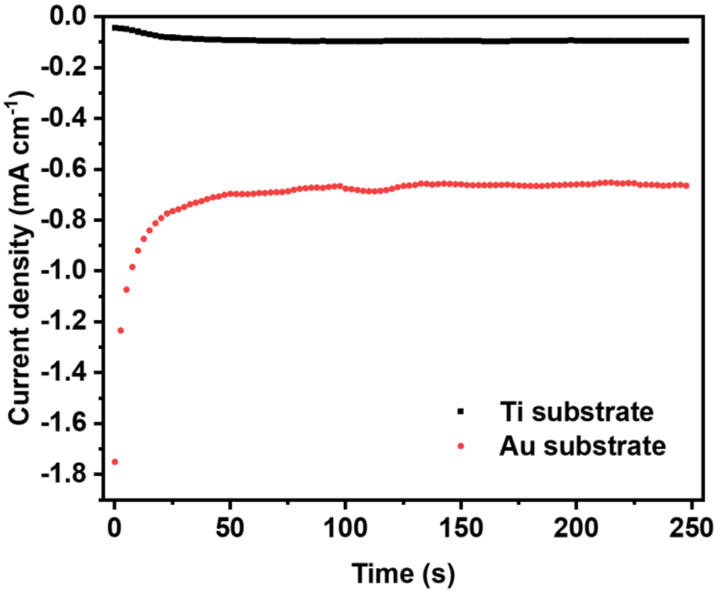

Fig. S6 Comparison of amperometry (*i*-*t*) curves for mesoporous Au deposition on (a) a Ti/Si substrate and (b) an Au substrate. The Au particles and films were deposited at -0.5 V for 250 s in the same area. The current density was normalized by the geometrical electrode area.

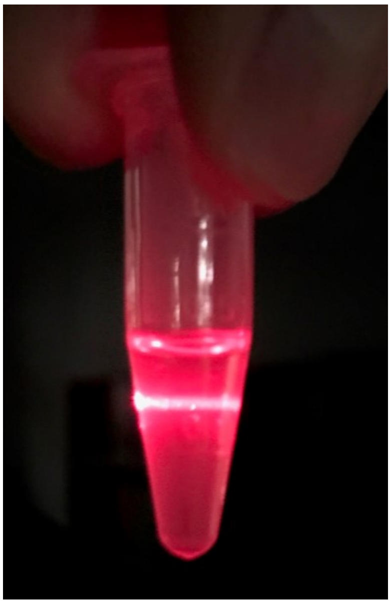

Fig. S7 Tyndall effect on colloidal HMAuNPs dispersed in ethanol. No precipitation was observed.

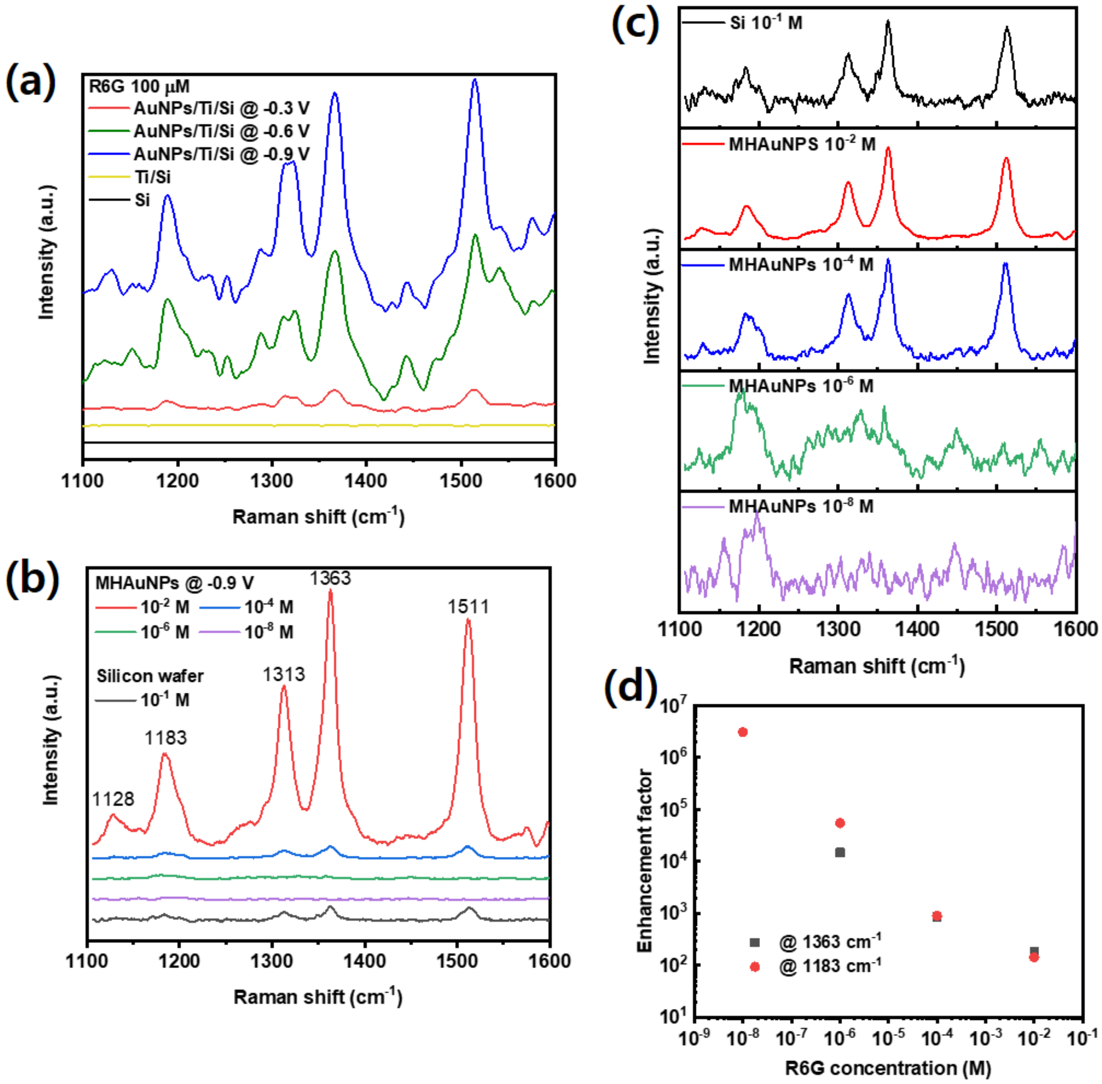

Fig. S8 (a) SERS spectra on MHAuNPs/Ti/Si substrates deposited at -0.3 V, -0.6 V, and -0.9 V for 1000 s, Ti/Si, and Si substrates. (b) SERS spectra on MHAuNPs/Ti/Si substrates with various R6G concentrations and (c) its normalized spectra of (b). (d) Enhancement factor-R6G concentration plots at 1363 cm^{-1} (black dots) and 1183 cm^{-1} (red dots).

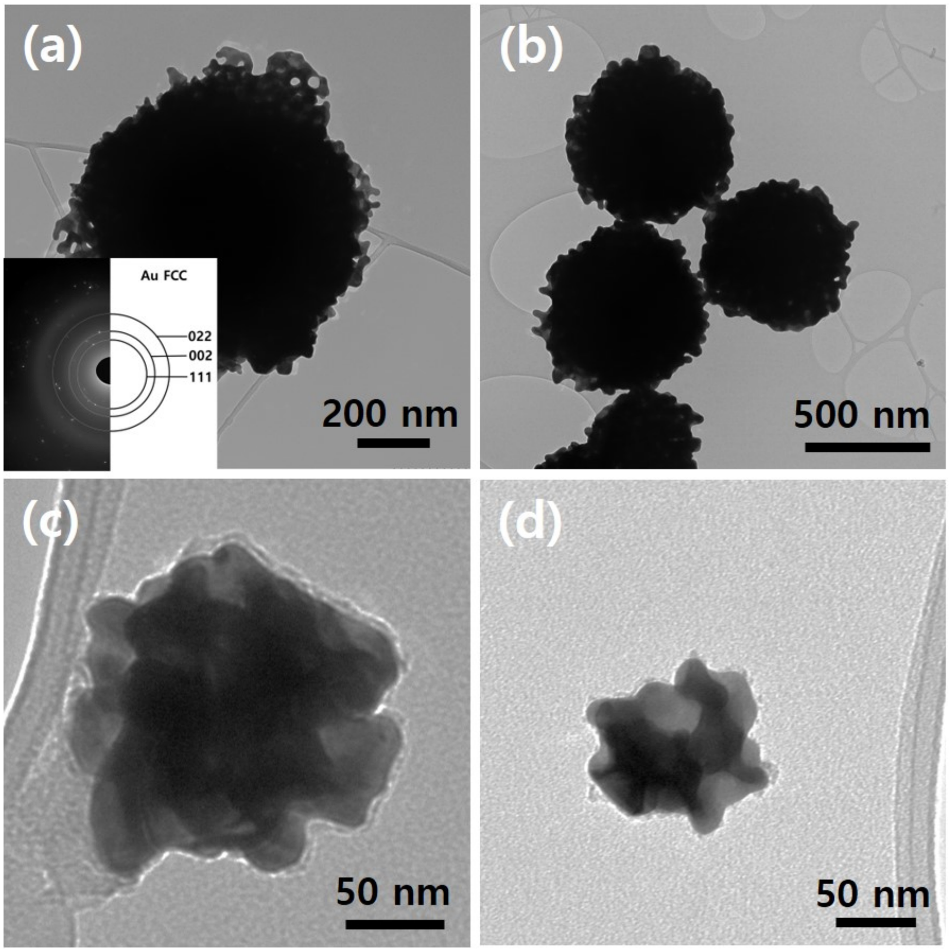

Fig. S9 TEM images of HMAuNPs deposited at (a, c, d) -0.3 V and (b) -0.9 V. Selected area electron diffraction patterns from one particle is shown as an inset. To clearly investigate the internal structure, we intentionally selected small sized HMAuNPs in **Fig. S9c-d**.

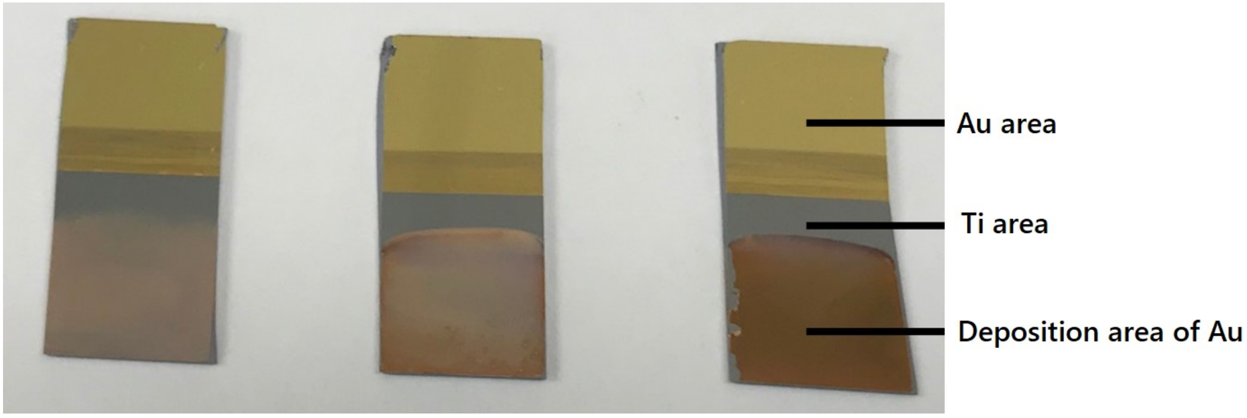

Fig. S10 Photographic images of MHAuNPs deposited at (left) -0.3 V, (middle) -0.6 V, and (right) -0.9 V for 1000 s on Ti/Si substrates.
